# Perforin gene *PRF1* c.900C> T polymorphism and HIV-1 vertical transmission

**DOI:** 10.1590/1678-4685-GMB-2018-0243

**Published:** 2019-11-14

**Authors:** Luisa Zupin, Vania Polesello, Anselmo Jiro Kamada, Rossella Gratton, Ludovica Segat, Louise Kuhn, Sergio Crovella

**Affiliations:** 1 Department of Medicine, Surgery and Health Sciences, University of Trieste, Trieste, Italy.; 2 Institute for Maternal and Child Health IRCCS “Burlo Garofolo”, Trieste, Italy.; 3 Departmento de Genética, Universide Federal de Pernambuco, Recife, PE, Brazil.; 4 Gertrude H. Sergievsky Center and Department of Epidemiology, Mailman School of Public Health, Columbia University, New York, NY, USA.

**Keywords:** PRF1, perforin, HIV-1 susceptibility

## Abstract

Perforin-1, a component of the immune system, is able to control Human Immunodeficiency Virus-1 (HIV-1) replication and could be involved in HIV-1 mother-to-child transmission (MTCT). This study aims at evaluating the role of the c.900C > T *PRF1* gene (encoding for perforin-1) polymorphism (rs885822) in HIV-1 MTCT. The *PRF1* c.900C > T polymorphism was genotyped in 331 children from Zambia using a Taqman probe on a Real Time PCR platform. The *PRF1* c.900C > T C/T genotype was more frequent among HIV-1 exposed but non-infected children than in HIV-1 positive cases, and the results were confirmed among children infected during breastfeeding. *PRF1* c.900C > T correlated with protection against HIV-1 MTCT, suggesting its role in HIV-1 vertical transmission.

Perforin-1 (pore forming protein) is a protein present in the granules of cytotoxic T lymphocytes (CTLs) and natural killer (NK) cells (Heintel *et al.*, 2002). In the presence of calcium, Perforin-1 polymerizes and forms channels in the target cell membrane through which other components of lytic granules including granzyme A, granzyme B and granulysin may enter in the cells (Shresta *et al.*, 1998; Stenger *et al.*, 1999). Therefore, Perforin-1 is one of the fundamental components of the death machinery of CTLs. CTLs possess anti-viral activity and in Human Immunodeficiency Virus-1 (HIV-1) infection they could concur in the control of viremia both during the initial and the persistent phases of infection (Musey *et al.*, 1997; Ogg *et al.*, 1998). The fraction of perforin-expressing HIV-1 specific CD8+ T cells inversely correlates with the peripheral blood CD4+ T cell count thus being a marker for disease progression (Shresta *et al.*, 1998).

Perforin-1 expression in *ex vivo* HIV-specific CD8+ T cells was described as higher in healthy controls compared to patients with uncontrolled viral replication, and an inverse correlation between perforin-1 expression in HIV-specific CD8+ T cells and viral load was observed (Migueles *et al.*, 2008; Hersperger *et al.*, 2010). Perforin-1 is encoded by the *PRF1* gene, located at 10q22, and one polymorphism, namely c.900C>T (rs885822), was previously associated with HIV-1 vertical transmission in a Brazilian population (Padovan *et al.*, 2011). In the present study, *PRF1* c.900C>T was analyzed in a population of HIV-1 exposed (infected and uninfected) children from Zambia in order to replicate previous findings, contributing to disclose its possible involvement in HIV-1 mother to child transmission (MTCT).

In this study, a subset of the population enrolled in the Zambia Exclusive Breastfeeding Study (ZEBS, Lusaka Zambia ClinicalTrials.gov Identifier: NCT00310726) was recruited. Briefly, ZEBS is a randomized clinical trial implied in the investigation of the relationship between the time of breastfeeding (i.e. exclusive breastfeeding up to 4 months, or breastfeeding with a median of 16 months) and the risk of HIV-1 transmission and child mortality. Between May 2001 and September 2004, 958 HIV-1 positive women were followed during pregnancy up to the delivery and until 24 months postpartum (PP) with their infants. Newborns were tested for HIV-1. All women were counseled to breastfeed to at least 4 months, and then half of them were randomized to stop breastfeeding and the other half to continue it. Women received only a single-dose nevirapine as prophylaxis to prevent HIV-1 MTCT.

For this analysis, 331 infants were selected, 22 had intrauterine (IU) HIV-1 transmission (defined as a positive polymerase chain reaction (PCR) result within 2 days of birth), 25 had intrapartum (IP) HIV-1 MTCT (defined as a positive PCR result within 42 days of birth with an earlier negative result), and 38 had postnatal (breastfeeding) HIV-1 MTCT (defined as a positive PCR results older than 42 days with a negative earlier result in a breastfed child), 246 were HIV-1 exposed but uninfected children (designed as HIV-). All women provided a written informed consent allowing children to participate in the study. All the experiments and procedures have been performed in accordance with ethical standards of the 1975 Declaration of Helsinki (7^th^ revision 2013) and the Ethics Committee of IRCCS Burlo Garofolo approved the research (protocol L-1106 1 May 2010).

DNA was extracted as reported by Segat *et al.* (2014). The *PRF1* polymorphism was detected using TaqMan SNPs genotyping C___1799201_10 assay and TaqMan® GTXpress Master Mix with the ABI7900HT Real Time PCR platform (Applied Biosystems - Life Technologies, Carlsbad, CA U.S.A.) following the manufacturer’s instructions.

The *PRF1* allele and genotype frequencies were calculated by direct counting. Fisher’s exact test was used for pairwise comparison of allele and genotypes. Logistic regression and Wald’s test were conducted to examine the association between polymorphism genotypes and the risk of HIV-1 MTCT. The statistical tests were performed with the free software R version 3.1.3 (R Core Team, 2018). Post-hoc power calculations were performed with G*Power software version 3.1.9.2 using post-hoc calculation employing Fisher’s exact test (Faul *et al.*, 2007).

The *PRF1* c.900C > T C/T genotype was more frequent among HIV- compared to HIV+ children, and was associated with decreased risk of acquiring HIV-1 infection (*p*=0.03, OR=0.47, CI=0.23-0.94; power=0.68; Table 1 and Table S1) also after adjustment for maternal CD4 cells count and HIV-1 plasma viral load (*p*=0.01, OR=0.40, CI=0.19-0.81; data not shown). When children were subdivided according to the route of transmission, C allele and C/T genotype correlated with protection towards HIV-1 MTCT in the group of PP infected children (C allele: *p*=0.02, OR=0.35, CI=0.11-0.90; power=0.64; and C/T genotype: *p*=0.01, OR=0.22, CI=0.04-0.74; power=0.50; Table 1 and Table S1) also after adjustment for maternal CD4 cells count and HIV-1 plasma viral load (*p*=0.009, OR=0.19, CI=0.05-0.66; data not shown).

**Table 1:** *PRF1* polymorphism allele genotype frequencies (and counts) in HIV-1 exposed but not infected children (HIV-) and HIV-1 infected children (HIV+) stratifying for timing of HIV-1 mother to child transmission (MTCT) in intrauterine (IU) intrapartum (IP) and postpartum (PP) groups.

Children	HIV+	IU	IP	PP	HIV-	HIV+ vs. HIV-	IU vs. HIV-	IP vs. HIV-	PP vs. HIV-
	n=85	n=22	n=25	n=38	n=246				
*PRF1*									
**c.900C > T rs885822**									
T	0.89 (151)	0.82 (36)	0.88 (44)	0.93 (71)	0.83 (410)	Ref.	Ref.	Ref.	Ref.
C	0.11 (19)	0.18 (8)	0.12 (6)	0.07 (5)	0.17 (82)	*p*=0.11; CI=0.35-1.09; OR=0.63	*p*=0.83; CI=0.43-2.55; OR=1.11	*p*=0.54; CI=0.23-1.68; OR=0.68	*p*=0.02; CI=0.11-0.90; OR=0.35
T/T	0.81 (69)	0.73 (16)	0.76 (19)	0.87 (34)	0.70 (171)	Ref.	Ref.	Ref.	Ref.
C/T	0.15 (13)	0.18 (4)	0.24 (6)	0.13 (3)	0.28 (68)	*p*=0.03; CI=0.23-0.94; OR=0.47	*p*=0.60; CI=0.15-2.05; OR=0.63	*p*=0.82; CI=0.25-2.18; OR=0.79	*p*=0.01; CI=0.04-0.74; OR=0.22
C/C	0.04 (3)	0.09 (2)	0.00 (0)	0.004 (1)	0.03 (7)	*p*=1.00; CI=0.17-4.82; OR=1.06	*p*=0.19; CI=0.28-17.83; OR=3.03	n.c.	*p*=1.00; CI=0.01-5.90; OR=0.72
HWE	χ^2^=4.48 p=0.03	χ^2^=3.33 p=0.07	χ^2^=0.46 p=0.49	χ^2^=4.86 p=0.03	χ^2^=0.01 *p*=0.94				

Our results partially agree with those of Padovan *et al.* (2011). In fact, both studies observed an increased frequency of c.900C>T T allele in the HIV-1 positive children group if compared to the group of HIV-1 exposed but not infected children. Our study found the c.900C>T T/T genotype to be more frequent among HIV+ respect to T/C genotype, while in the study of Padovan *et al.* (2011) T/T was more frequent compared to C/C homozygous genotype. The study of McIllroy *et al.* (2006) also analyzed this *PRF1* gene polymorphism in a cohort of French HIV+ sero-converters. They observed that *PRF1* c.900C>T polymorphism seemed not to alter the amino acidic sequence of perforin-1 protein and was not associated with HIV-1 infection or progression. The different mode of HIV-1 transmission and different ethnic genomic background could account for the divergent results.

In the current study, we observed an association of *PRF1* polymorphism with the susceptibility to HIV-1 in the HIV+ group, but intriguingly, it was confirmed only in the infants that presented PP MTCT, thus indicating a protective effect of the variants at birth and not during the pregnancy or the delivery.

The functional effect of this polymorphism on the protein and its possible influence in HIV-1 vertical transmission were not yet reported. A hypothesis suggested by Padovan *et al.* (2011) indicated the *PRF1* c.900C>T polymorphism as exerting possible effects on protein expression, which might in turn influence NK functionality. Indeed, the NK response plays a pivotal role in preventing HIV-1 vertical transmission, as a higher HIV-1 specific NK response was found in HIV-1-infected non transmitter mothers and exposed-uninfected children compared to transmitter mothers and exposed-infected children (Tiemessen *et al.*, 2009). However, this speculation should be confirmed by functional analysis, which have not been performed in our study due to the fact that the sole biological material available were dried blood spots.

We are aware that the small sample size of our population could have influenced the statistical analysis, especially in the subgroups classified according to the route of MTCT. However, the quite high power of the statistically significant associations allows us to be confident about the statistical relevance of the results. We also decided to not perform corrections for multiple testing in order to unravel all the possible associations that could be significant, especially in an infectious disease where role of genetic polymorphisms should be small, and since after applying multiple test corrections our significance will be lost.

Another point that should be taken into account is the MTCT, as even when the viral HIV-1 RNA is undetectable, the risk MTCT still exists (see for example Reliquet *et al.*, 2014). However, the modern test for virologic diagnosis did not reveal possible infections, so possibly creating a bias in our analysis.

Considering our findings and the comparison with the two other studies analyzing the role of *PRF1* variants in the context of HIV-1 infection, further association studies in populations of different ethnic backgrounds are necessary to disclose the effective role of perforin-1 in HIV-1 MTCT susceptibility.

## Supplementary material

The following online material is available for this article:

Table S1 The results from power analysis (Fisher's exact test).
