# Supplementary material for: Perforin gene PRF1 c.900C> T polymorphism and HIV-1 vertical transmission
Source: Genet Mol Biol. 2019 Nov 14;42(3):574–7. doi: 10.1590/1678-4685-GMB-2018-0243 (PMC6905447; doi:10.1590/1678-4685-GMB-2018-0243)
Supplement: Supplementary file 1 [file 1415-4757-GMB-42-3-2018-0243-20190911-suppl.pdf]

Supplementary Material to “Perforin gene *PRF1* c.900C>T polymorphism and HIV-1 vertical transmission”

Table S1 - The results from power analysis (Fisher’s exact test).

**Children**	**HIV+**	**IU**	**IP**	**PP**	**HIV-**	**HIV+ vs. HIV-**	**IU vs. HIV-**	**IP vs. HIV-**	**PP vs. HIV-**
	n=85	n=22	n=25	n=38	n=246				
***PRF1* c.900C>T rs885822**									
T	0.89 (151)	0.82 (36)	0.88 (44)	0.93 (71)	0.83 (410)				
C	0.11 (19)	0.18 (8)	0.12 (6)	0.07 (5)	0.17 (82)	Power = 0.45<br>Actual α = 0.04	Power = 0.05<br>Actual α = 0.04	Power = 0.10<br>Actual α = 0.04	Power = 0.64<br>Actual α = 0.04
T/T	0.81 (69)	0.73 (16)	0.76 (19)	0.87 (34)	0.70 (171)				
C/T	0.15 (13)	0.18 (4)	0.24 (6)	0.13 (3)	0.28 (68)	Power = 0.68<br>Actual α = 0.04	Power = 0.13<br>Actual α = 0.04	Power = 0.04<br>Actual α = 0.03	Power = 0.50<br>Actual α = 0.04
C/C	0.04 (3)	0.09 (2)	0.00 (0)	0.004 (1)	0.03 (7)	Power = 0.06<br>Actual α = 0.03	Power = 0.24<br>Actual α = 0.02		

HIV- = HIV-1 exposed but not infected children
HIV+ = HIV-1 infected children
IU = intrauterine HIV-1 mother to child transmission
IP = intrapartum HIV-1 mother to child transmission
PP = postpartum HIV-1 mother to child transmission
